# *H. pylori* negative gastric MALT lymphoma with API2-MALT1 translocation treated by endoscopic submucosal dissection: A case report

**DOI:** 10.1097/MD.0000000000024371

**Published:** 2021-04-09

**Authors:** Tomomitsu Tahara, Noriyuki Horiguchi, Tsuyoshi Terada, Dai Yoshida, Masaaki Okubo, Kohei Funasaka, Yoshihito Nakagawa, Tomoyuki Shibata, Naoki Ohmiya

**Affiliations:** a Third Department of Internal Medicine, Kansai Medical University, Osaka; b Department of Gastroenterology, Fujita Health University School of Medicine, Toyoake, Japan.

**Keywords:** API2-MALT1, endoscopic submucosal dissection, gastric MALT lymphoma, *H. pylori* negative

## Abstract

**Rationale:**

API2-MALT1 positive gastric mucosa-associated lymphoid tissue (MALT) lymphomas are considered to have favorable prognosis. We report a case of API2-MALT1 positive gastric MALT lymphoma, treated by endoscopic submucosal dissection (ESD).

**Patient concerns:**

A 51-year-old man underwent esophagogastroduodenoscopy (EGD) for the annual health checkup examination.

**Diagnoses:**

The EGD showed a reddish depressed lesion with small reddish spots in the lower gastric body. There was no endoscopic atrophy in the entire stomach and *Helicobacter pylori* (*H. pylori*) serum test was negative. Infiltration of small lymphocytes was shown in the gastric tissues obtained by the endoscopic biopsy. The fluorescence in situ hybridization using the biopsy samples confirmed the presence of genetic translocation of API2-MALT1, suggesting that the lesion is API2-MALT1 positive MALT lymphoma.

**Interventions:**

Since endoscopic ultrasound suggested that the lesion was localized within the lamina propria mucosae, we performed ESD to achieve the en bloc resection of the lesion.

**Outcomes:**

Conclusive diagnosis of gastric MALT lymphoma was made based on the resected specimen. Lateral and vertical margins were negative. No lymphoma cells were detected using endoscopic biopsy after 5 years.

**Lessons:**

Our report suggests that ESD can be considered as alternative treatment for API2-MALT1 positive gastric MALT lymphoma if the lesion was localized within the gastric mucosa.

## 1 Introduction

Extranodal marginal zone lymphoma of mucosa-associated lymphoid tissue (MALT) lymphoma is a non-Hodgkin lymphoma derived from marginal zone B-cells, which occurs in a number of extranodal organs, including the gastrointestinal tract, lung, salivary gland, thyroid, ocular adnexa, liver, or skin.^[1]^ The stomach is the most frequent site for MALT lymphoma.^[1,2]^*Helicobacter pylori* (*H. pylori*) plays a crucial role in the development of gastric MALT lymphoma. Majority of MALT lymphomas are *H. pylori* infection positive and are quite sensitive to *H. pylori* eradication.^[3,4]^ Genetic abnormalities are also common in MALT lymphomas and various chromosomal translocations have been described.^[1]^ In gastric MALT lymphoma, t(11;18)/API2-MALT1 is the most frequent translocation detected in 20% of cases.^[5]^ The API2-MALT1 positive MALT lymphomas are thought to be associated with absence of *H. pylori* infection. Moreover, the API2-MALT1 positive cases are resistant to *H. pylori* eradication.^[5]^ For the second-line oncological treatment for cases resistant to *H. pylori* eradication, radiotherapy is highly effective in localized cases,^[3,6]^ which may be also considered for the API2-MALT1 translocation positive cases. However, it has been also reported that API2-MALT1 positive gastric MALT lymphomas showed rather favorable prognosis such as no progression of clinical stage, high-grade transformation, or chromosomal aberrations.^[7,8]^ Here, we show a case of *H. pylori* negative gastric MALT lymphoma with API2-MALT1 translocation successfully treated by endoscopic submucosal dissection (ESD).

## 2 Case report

### 2.1 Patient concerns

The patient has provided informed consent for publication of the case. A 51-year-old man underwent esophagogastroduodenoscopy (EGD) for the annual health checkup examination. He had no remarkable medical history and no abnormality was observed on physical examination.

### 2.2 Diagnosis

**Figure 1:**
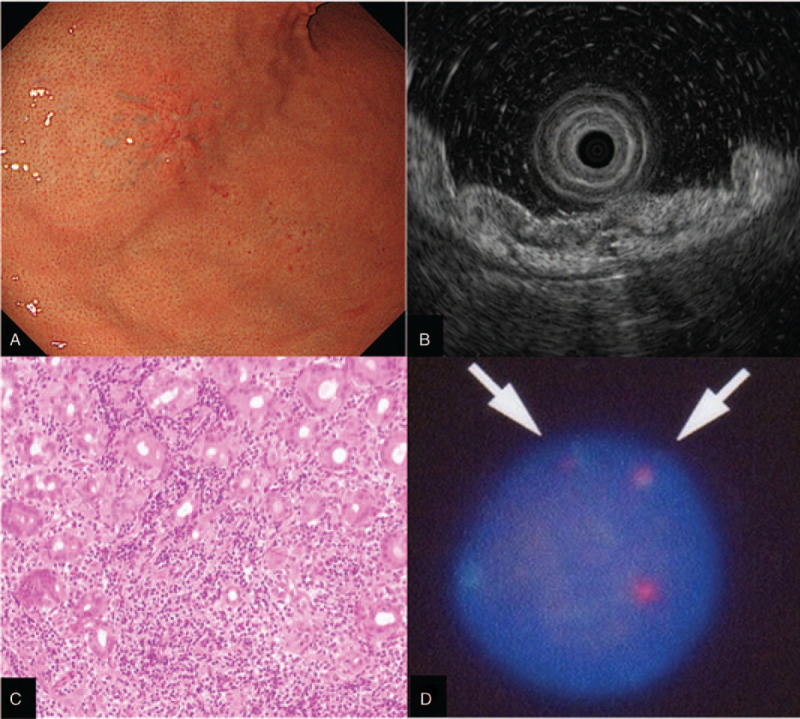
Representative endoscopic photograph of the gastric lesion. (A) A reddish depressed lesion was shown in the lower gastric body. Reddish spots were also shown in the surrounding mucosa of the main lesion. (B) Endoscopic ultrasound. Low echoic shadows were observed in lamina propria mucosae, suggesting that the lesion was localized within the gastric mucosal layer. (C) Infiltration of small lymphocytes was shown in the gastric tissues obtained by the endoscopic biopsy. (D) The fluorescence in situ hybridization (FISH) using the endoscopic biopsy specimens confirmed the presence of genetic translocation of API2-MALT1 (white arrow).

EGD showed a reddish depressed lesion in the lower gastric body. Small reddish spots were also observed in surrounding mucosa of the main lesion (Fig. 1(A)). There was no endoscopic atrophy in the entire stomach and the helicobacter pylori (*H. pylori*) serum test was negative, suggesting that this patient has no previous history of *H. pylori* infection. The endoscopic ultrasound (EUS) showed low echoic shadow in the lamina propria mucosae (Fig. 1(B)). Infiltration of small lymphocytes was shown in the gastric tissues obtained by the endoscopic biopsy (Fig. 1(C)). The fluorescence in situ hybridization (FISH) using the biopsy sample confirmed the presence of genetic translocation of API2-MALT1 (Fig. 1(D)). The lesion was considered as API2-MALT1 positive MALT lymphoma.

### 2.3 Interventions

**Figure 2:**
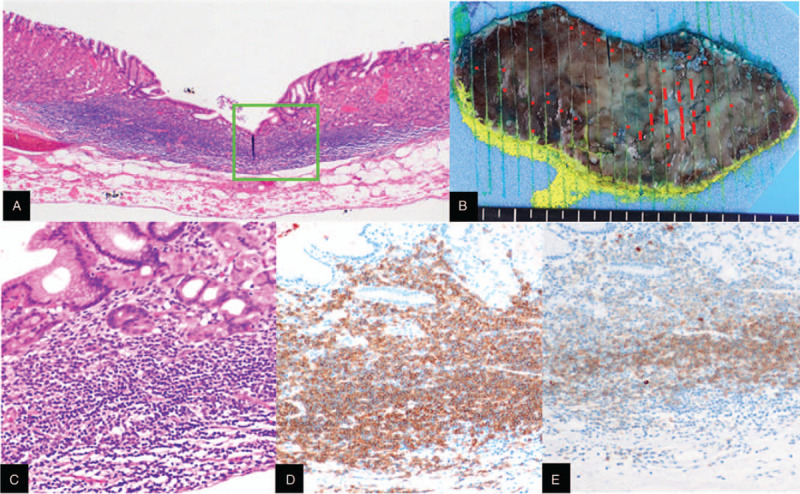
Histopathological examination of resected specimen. (A) Low power view of Hematoxylin and Eosin staining. A light green box indicates the area shown in (C), (D), and (E). (B) Schematic view of ESD specimen showing lateral extension of MALT lymphoma. Lymphoma cells were interspaced within resected specimen (red lines) but lateral and vertical margins were negative. (C) Microscopic examination of Hematoxylin and Eosin staining. Dense infiltration of small lymphocytes was shown predominantly in lamina propria mucosae. (D) and (E) Immunostaining for the CD20 and CD79a, respectively. Infiltrated lymphocytes showed positive immunostaining for the CD20 and CD79a. A conclusive MALT was made. MALT = mucosa-associated lymphoid tissue.

Because the case was considered resistant to *H. pylori* eradication and radiotherapy would be possible oncological treatment.^[3,6]^ However, API2-MALT1 positive gastric MALT lymphomas can be also considered as favorable prognosis.^[7,8]^ Since EUS suggested that the lesion was localized within the lamina propria mucosae, we performed ESD to achieve the en bloc resection of the lesion. Microscopic examination showed dense infiltration of small lymphocytes predominantly in the lamina propria mucosae (Fig. 2(A) and (C)). Since the infiltrated lymphocytes showed positive immunostaining for the CD20, and CD79a (Fig. 2(D) and (E)), a conclusive diagnosis of gastric B-cell lymphoma of the mucosa-associated lymphoid tissue (MALT) type was made. The microscopic examination indicated that lymphoma cells were inter-speared within the resected specimen (Fig. 2(B), red lines) but lateral and vertical margins were negative. No other malignancies were also observed using computed tomography (CT) and PET/CT.

### 2.4 Outcomes

No lymphoma cells were detected using endoscopic biopsy after 5 years.

## 3 Discussion

The API2-MALT1 positive gastric MALT lymphomas are resistant to *H. pylori* eradication^[5]^ but have rather favorable prognosis. The API2-MALT1 positive gastric MALT lymphomas show no progression of clinical stage, high-grade transformation, or chromosomal aberrations.^[7,8]^ A “watch and wait” strategy is sometimes considered for the cases with persistent histological lymphoma without progression.^[9]^ On the other hand, radiotherapy is also effective for localized cases,^[3,6]^ which can be considered for cases resistant to *H. pylori* eradication. However, standard doses of 30 Gy of radiotherapy (1.5 Gy/day, 5 days/week) requires at least 1 month of hospitalization.^[10,11]^ Risk of both the short-term and long-term side effects of the radiotherapy should be also considered.^[12,13]^ In addition, radiotherapy may lead to an increased risk of second malignancies. The patient in our case report did not agree to receive radiotherapy because of the longer hospitalization and risk of side effects. We carefully explained the result of all examination and suggest the possibility of ESD to remove the lesion. The ESD was completed within 3 hours and the patients were discharged after 5 days. No adverse events associated with ESD were also noted including bleeding, perforation, and aspiration pneumonia. The patients showed no recurrence of lymphoma after 5 years, suggesting that ESD would be good option for *H. pylori* negative gastric MALT lymphoma with API2-MALT1 translocation. However, to perform ESD for gastric MALT lymphoma, careful treatment decision should be made according to the depth of the lesion. In our case, the lesion was appeared as superficial depressed lesion. The EUS finding also suggests that the lesion is localized within the lamina propria mucosae. We therefore decided to perform ESD to remove the lesion. The en bloc specimen obtained by the ESD enabled us to precise histological evaluation. Our case suggests that the ESD would be useful for both diagnosis and treatment.

In our knowledge, this is the first reporting the ESD for the gastric MALT lymphoma. Our case indicates that ESD can be considered as the alternative treatment for *H. pylori* negative gastric MALT lymphoma with API2-MALT1 translocation although careful treatment decision and follow up would be needed.

## Author contributions

**Conceptualization:** Tomomitsu Tahara, Noriyuki Horiguchi.

**Data curation:** Tomomitsu Tahara, Noriyuki Horiguchi, Tsuyoshi Terada, Dai Yoshida, Masaaki Okubo.

**Investigation:** Tomomitsu Tahara.

**Supervision:** Kohei Funasaka, Yoshihito Nakagawa, Tomoyuki Shibata, Naoki Ohmiya.

**Writing – original draft:** Tomomitsu Tahara.

**Writing – review & editing:** Tomomitsu Tahara.
